# Single incision surgical approach for the release of lacertus syndrome and cubital tunnel syndrome

**DOI:** 10.1007/s00264-025-06494-4

**Published:** 2025-03-17

**Authors:** Mohammed Muneer, Salwa Al-Maraghi

**Affiliations:** 1 Hamad Medical Corporation, Doha, Qatar; 2 Qatar University, Doha, Qatar; 3 Qatar University, Doha, Qatar

**Keywords:** Cubital tunnel syndrome, Lacertus syndrome- lacertus fibrosus -multiple compression neuropathy.

## Abstract

**Background:**

understanding the concept of multiple compression neuropathy syndrome has recently evolved, leading to better clinical assessment and evaluation. However, decompression of the involved nerves might require multiple incisions. Concomitant compression neuropathy, such as Lacertus Syndrome (LS) and cubital tunnel syndrome, is not uncommon. The traditional approach for releasing both nerves encompasses two separate surgical incisions. Minimazing surgical incisions is essential for postoperative scar management and nerve gliding. In this paper we describe a single surgical incision for releasing both compressions.

**Surgical technique:**

To release the Lacertus Fibrosis using the classical surgical incision for cubital tunnel syndrome, an incision is made between the medial epicondyle and olecranon. After reaching the brachial fascia, the skin and subcutaneous tissue are raised as a one flap off the fascia. The lacertus fibrosis, identified as a thick rectangular or trapezoid stracture attached to the brachial fascia, is then incised to expose the median nerve beneath it.

**Conclusion:**

As we advance towards the concept of multiple compression neuropathy, it is crucial to minimize surgical incisions to reduce pain, wound breakdown, scar formation, traction neuropathy, neuroma formation, and unsatisfactory aesthetic outcomes.

**Supplementary Information:**

The online version contains supplementary material available at 10.1007/s00264-025-06494-4.

## Introduction

Patients may experience compression syndromes affecting multiple nerves in the same arm. This occurs when decompression of one nerve does not alleviate symptoms, and treatment outcomes are unsatisfactory due to the presence of additional nerve compressions [1, 2]. Multiple compression neuropathy syndrome is commonly seen in the upper limb. Recent studies have highlighted the varying prevalence of concomitant conditions such as carpal tunnel syndrome, lacertus syndrome, and cubital tunnel syndrome, underscoring the frequent overlap among these disorders [2–5].

The lacertus notch on the forearm’s medial side serves as a clinical marker for lacertus syndrome and potentially indicates concurrent cubital tunnel syndrome [2]. Constriction at the lacertus fibrosus may contribute significantly to the frequent occurrence of both ulnar and median neuropathies at the elbow.

Classically, surgical intervention for releasing ulnar nerve entrapment in the cubital tunnel and median nerve entrapment by the lacertus fibrosus involves two separate incisions: one between the medial epicondyle and olecranon and another proximally on the forearm, either transversely or obliquely. However, Multiple surgical incisions might elevate the risk of scar-related neuropathic pain and traction neuropathy due to perineural scarring. Additionally, these incisions restrict the natural gliding movement of the nerve, which can prolong recovery time and delay a patient’s return to normal activities [6].

Therefore, we propose a single-incision approach that offers advantages such as reducing the risk of nerve scaring, traction neuropathy, and painful scar. Additionally, it yields better cosmetic outcomes with a concealed scar and improves exposure for identifying anatomical variations of the lacertus fibrosus during median nerve release.

## Surgical technique

**Fig. 1:**
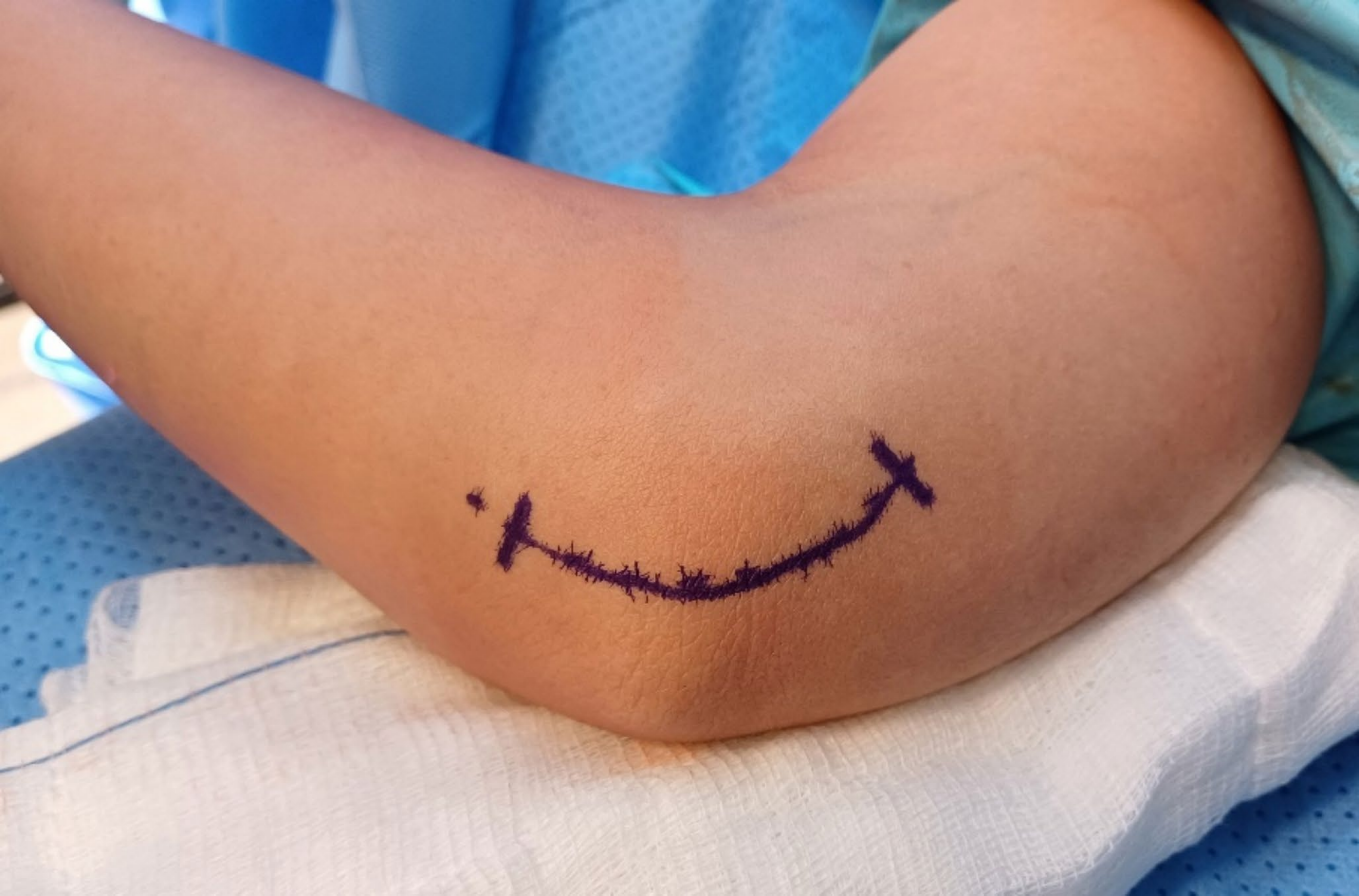
Single- incision approach for decompression of Cubital tunnel syndrome and Lacertus syndrome using the classical surgical incision for cubital tunnel syndrome, an incision is made between the medial epicondyle and olecranon

**Fig. 2:**
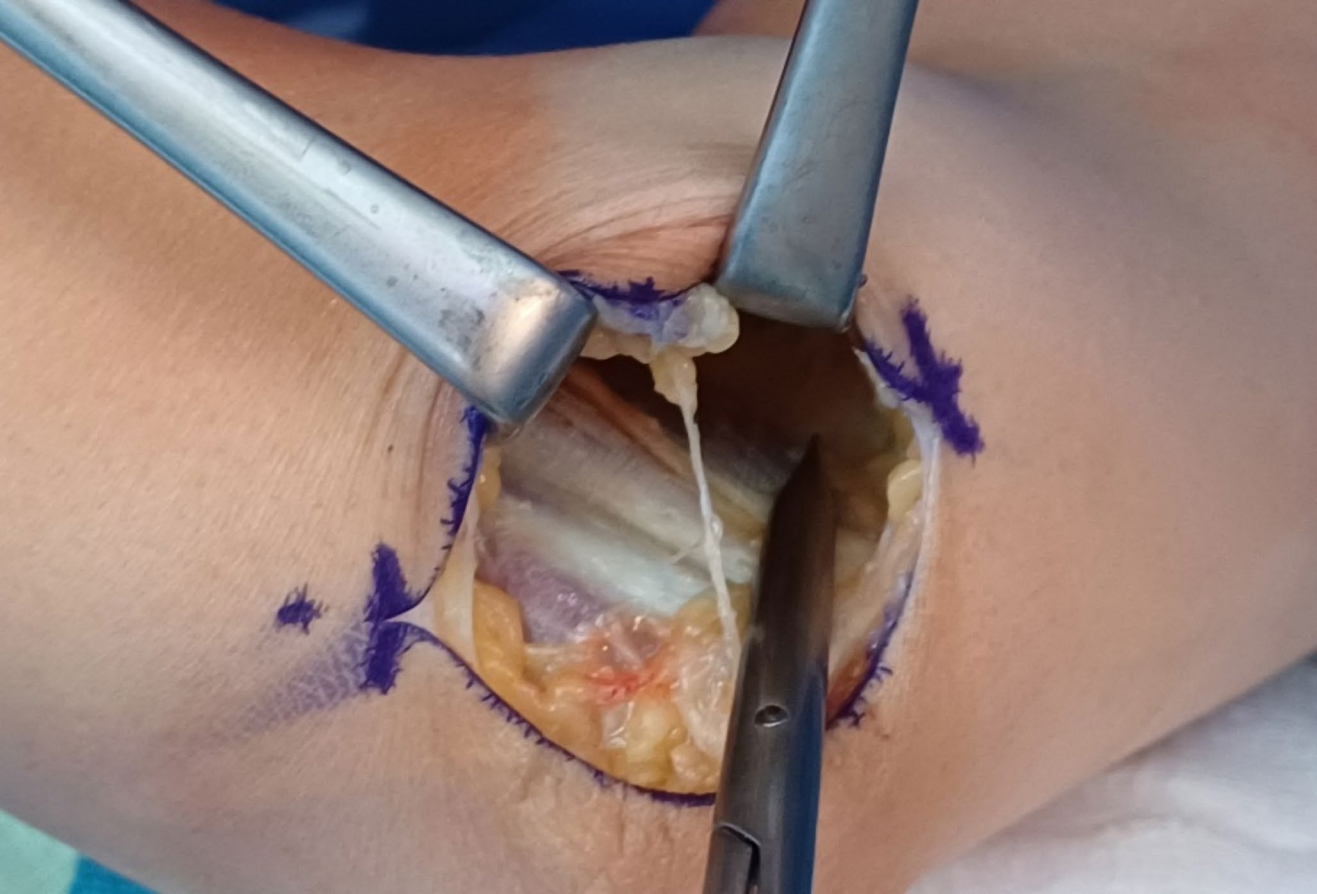
Identifying lacertus fibrosus through the single incision surgical approach

**Fig. 3:**
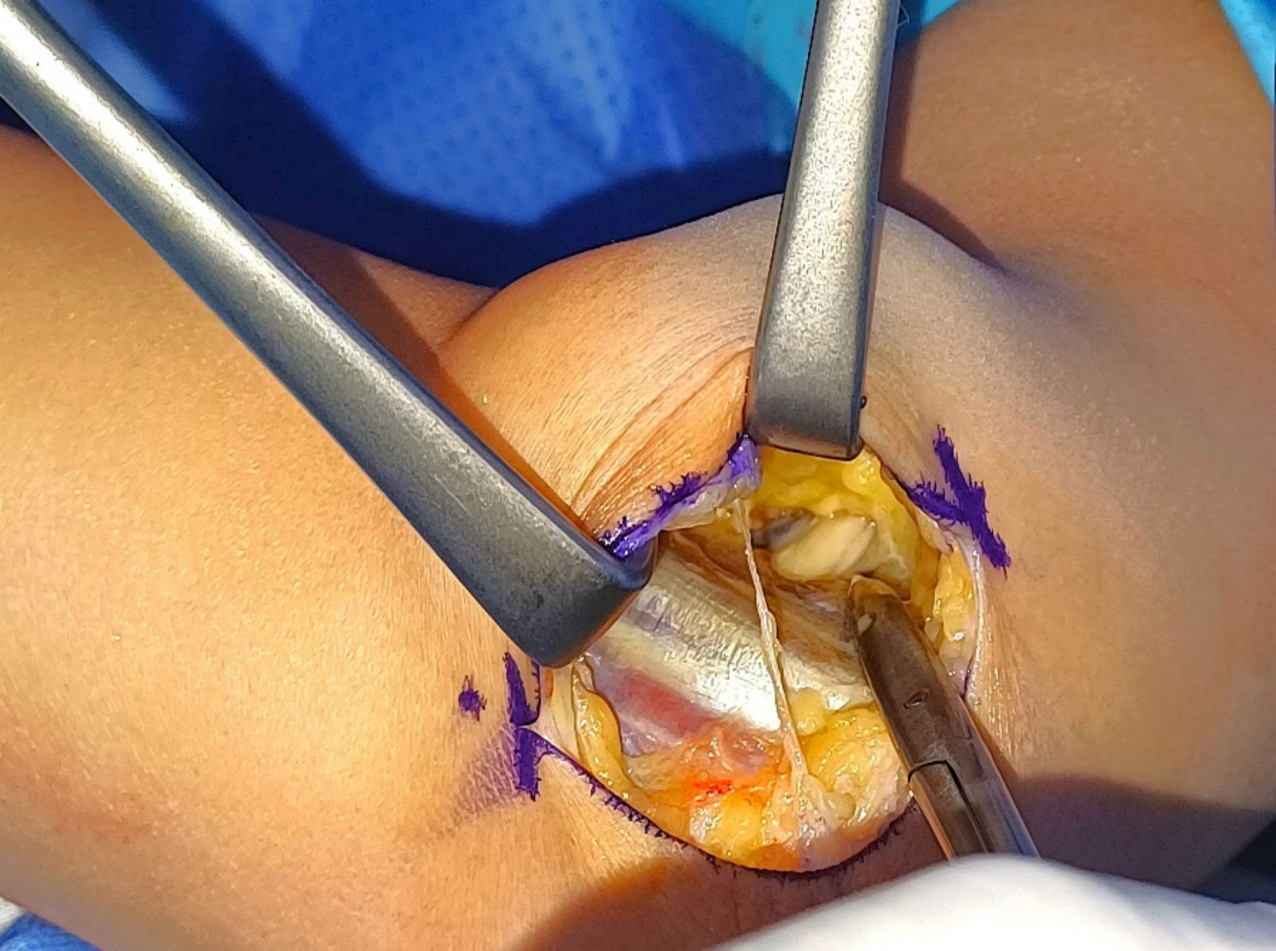
Identifying the median nerve after releasing lacertus fibrosus

**Fig. 4:**
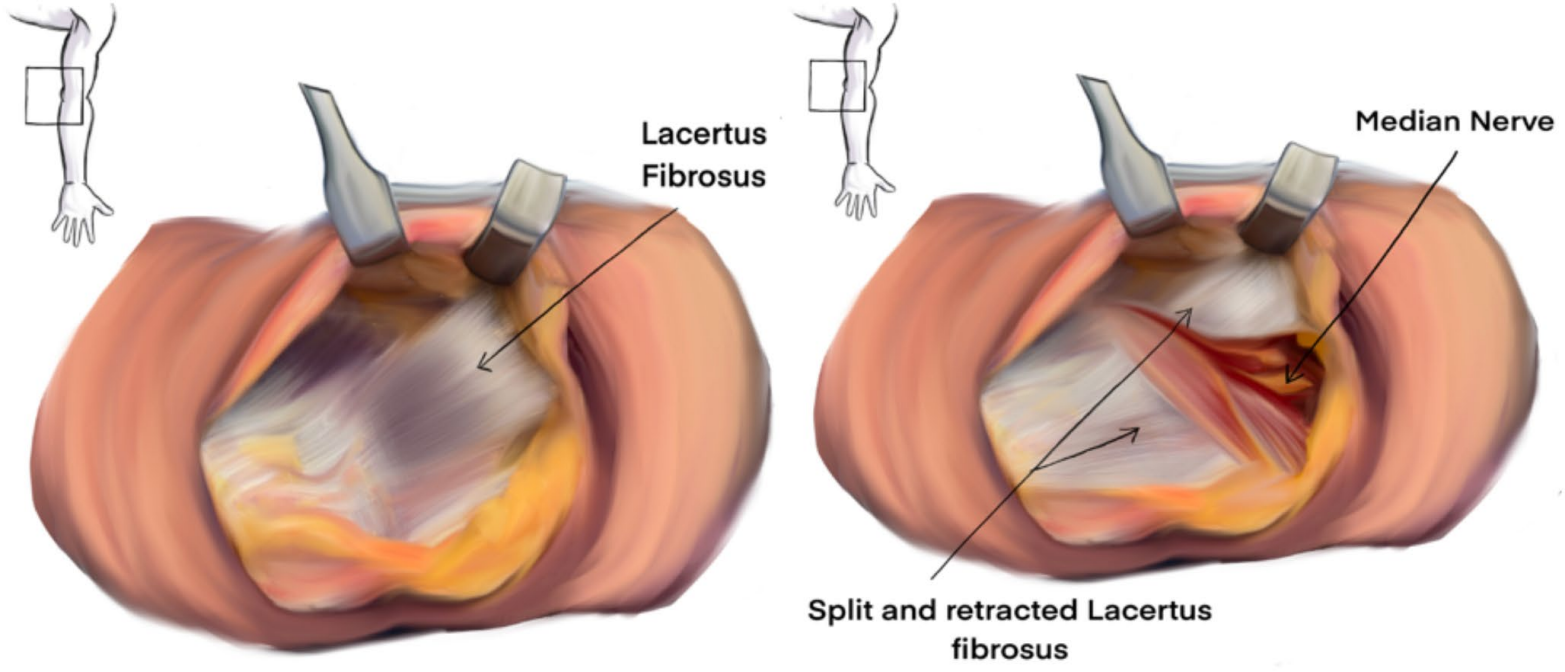
Illustration of the surgical technique: an incision is made between the medial epicondyle and olecranon. After reaching the brachial fascia, the skin and subcutaneous tissue are raised as one flap off the fascia. The lacertus fibrosis is identified and incised to expose the median nerve beneath it

To release the Lacertus Fibrosis using the classical surgical incision for cubital tunnel syndrome, an incision is made between the medial epicondyle and olecranon (Fig. 1). After reaching the brachial fascia, the skin and subcutaneous tissue are raised as a one flap off the fascia. The lacertus fibrosis, identified as a thick rectangular or trapezoid structure attached to the brachial fascia (Fig. 2), is then incised to expose the median nerve beneath it (Figs. 3 and 4).

## Discussion

The concept of multiple compression syndrome in the upper limb has gained significant attention in recent years, drawing interest from peripheral nerve surgeons worldwide. Managing concurrent nerve compressions remains a challenging task [1–3].

Patients may experience compression syndromes affecting different nerves in the same arm. This is evident when decompression of one nerve fails to relieve symptoms, and treatment outcomes are unsatisfactory due to the presence of another nerve compression [2]. As the understanding of multiple compression syndrome develops, it is becoming increasingly important to minimize the number of surgical incisions when addressing multiple nerve compressions. Multiple incisions, on the other hand, lead to scar formation, which can increase the risk of perineural scarring, limiting nerve glide and potentially causing traction neuropathy.

Perineural scarring, and the resulting traction neuropathy, have long been considered complications of nerve decompression surgery. Nerve tethering at the surgical site remains the primary cause of symptoms associated with perineural scarring. This condition is particularly challenging to manage. Reports suggest that compression symptoms persist in 40–90% of revision procedures, with approximately 20% of patients requiring a third surgery [6].

Furthermore, skin scarring can lead to structural stiffness, reduced elasticity, and hypersensitivity, all of which prolong post-operative rehabilitation. Thus, careful planning of surgical skin incisions is crucial when treating patients with multiple compression syndrome.

The concurrent lacertus syndome and cubital tunnel syndrome is not uncommon. However, it might be missed oweing to the close anatomical relation. The diagnosis of concurrent lacertus syndome and cubital tunnel syndrome is clinical due to limitations in electromyographic diagnosis and delayed diagnosis. The Hagert clinical triad, lacertus notch sign, W sign, lacertus antagonist test and taping help accurate diagnosis [7, 8]. Lacertus notch, an anatomical contour deformity on the anteromedial aspect of the proximal forearm that represent a pivotal physical sign for diagnosis of the concurrent lacertus syndrome and Cubital tunnel compression.

In conclusion, single-incision approach for releasing cubital tunnel syndrome and lacertus fibrosus offers advantages such as reducing the risk of nerve scaring, traction neuropathy, and painful scar. Additionally, it yields better cosmetic outcomes with a concealed scar.

## Electronic supplementary material

Below is the link to the electronic supplementary material.

Supplementary Material 1

## Data Availability

No datasets were generated or analysed during the current study.
