# Supplementary material for: Single incision surgical approach for the release of lacertus syndrome and cubital tunnel syndrome
Source: Int Orthop. 2025 Mar 17;49(6):1421–5. doi: 10.1007/s00264-025-06494-4 (PMC12075323; doi:10.1007/s00264-025-06494-4)
Supplement: Supplementary file 1 — Supplementary Material 1 [file 264_2025_6494_MOESM1_ESM.doc]

**Cover letter**

Date 27/01/2025

Dear Chief Editor,

I wish to submit an original research article entitled “**Single Incision Surgical Approach for the Release of Lacertus Syndrome and Cubital Tunnel Syndrome**” for consideration by the International Orthopedics. We are very interested to contribute in the “special Issue on Nerve Compression Syndromes" in International Orthopedics.

I confirm that this work is original and has not been published elsewhere, nor is it currently under consideration for publication elsewhere.

In this paper, I am describing Single Incision Surgical Approach for the Release of Lacertus Syndrome and Cubital Tunnel Syndrome that offers advantages such as reducing the risk of nerve scaring, traction neuropathy, and painful scar.

We have no conflicts of interest to disclose.

Please address all correspondence concerning this manuscript to me at mmyousif78@yahoo.com.

Thank you for your consideration of this manuscript.
